# Pneumocephalus and Catatonia in a Patient With Persistent Drain Dysfunctions and Infections After a Hydrocephalus due to an Aquaduct Stenosis: A Case Report

**DOI:** 10.7759/cureus.9440

**Published:** 2020-07-28

**Authors:** Sjaak Pouwels, Lowieke Vis, Dharmanand Ramnarain, Anne Rutten

**Affiliations:** 1 Intensive Care Medicine, Elisabeth-Tweesteden Hospital, Tilburg, NLD; 2 Cardiology, Amphia Hospital, Breda, NLD

**Keywords:** hydrocephalus, pneumocephalus, catatonia, aquaduct stenosis, drain dysfunction, infection

## Abstract

The combination of catatonia and pneumocephalus is very rare and literature is very scarce on this topic. Here, we report a patient with pneumocephalus and catatonia after persistent drain dysfunctions and infections after a hydrocephalus due to an aquaduct stenosis. The combination of a catatonic syndrome and pneumocephalus in neurosurgical and intensive care practice is a rare phenomenon. One should consider it in patients after complicated neurosurgery with pneumocephalus.

## Introduction

Pneumocephalus is a condition that is seen after several neurosurgical procedures, in particular intracranial surgery of the posterior fossa [1-2]. Its incidence can vary and might be related to the intracranial surgical procedures performed in sitting or half-sitting position [1-2]. Pneumocephalus is also seen after surgical drainage of subdural haematomas, after (neuro) trauma, and even after sinus surgery [3-5]. Clinical symptoms can vary and if a tension pneumocephalus occurs it needs urgent treatment [3].

Kahlbaum first described catatonia in 1870s as a complex syndrome that comprised bizarre behavior, impaired volition, and vegetative abnormalities [6-7]. Difficulties arise when clinicians try to measure and treat catatonia as its original classification has over 40 signs and symptoms [8-9]. These signs may be summarized in four groups [6-7]: pure motor signs (e.g., posturing, rigor, immobility), disturbances of volition (e.g., ambitendence, negativism, automatic obedience), inability to suppress complex motor activities (e.g., stereotypies, rituals, echophenomena), and autonomic instability (e.g., tachycardia, hyperthermia) [6-7]. Also the last year’s changes have occurred in the symptomatology and also different classifications are present in the literature, which indicate the fluidity of the boundaries of the clinical concept catatonia [10]. There is no consensus on what signs and symptoms constitute a catatonic syndrome, or how many different catatonic syndromes there are [8]. This provides an explanation why catatonia-rating scales are so different from each other [10-11]. The Bush-Francis Catatonia Rating Scale (BFCRS) was the first instrument constructed for the systematic, standardized, and quantifiable examination of catatonia using operationally defined signs and symptoms [10-11].

Here, we report a patient with pneumocephalus and catatonia after persistent drain dysfunctions and infections after a hydrocephalus due to an aquaduct stenosis. We assessed the catatonia signs and symptoms using the BFCRS.

## Case presentation

Here we report a 68-year-old male with traumatic brain injury (due to a car accident) and cognitive dysfunction. Since 30 years he had a hydrocephalus for which a ventriculo-atrial drain was placed and six years after placement it got infected. This was treated successfully with antibiotics at that time. His medical history reported chronic obstructive pulmonary disease (COPD) Gold 2, multiple transient ischemic attacks (TIAs), cerebrovascular accidents (CVAs), paroxysmal atrial fibrillation (for which he had anticoagulants), and a bleeding in the basal ganglia.

His ventriculo-atrial drain was revised due to drain dysfunction and was converted to a ventriculoperitoneal drain. Due to persistent recurrent hydrocephalus on the CT scan and cognitive problems, a second drain revision was conducted one month later. Unfortunately, this drain was positioned extraperitoneally and was revised for a third time. Directly after the third drain revision, the patient developed a fever due to a drain infection and therefore an external ventricular drain was placed and antibiotic treatment was started. The bacterial cultures of the cerebrospinal fluid (CSF) remained negative. During the treatment for the drain infection, the patient developed respiratory problems due to pulmonary embolism, which were treated with anticoagulants.

**Figure 1:**
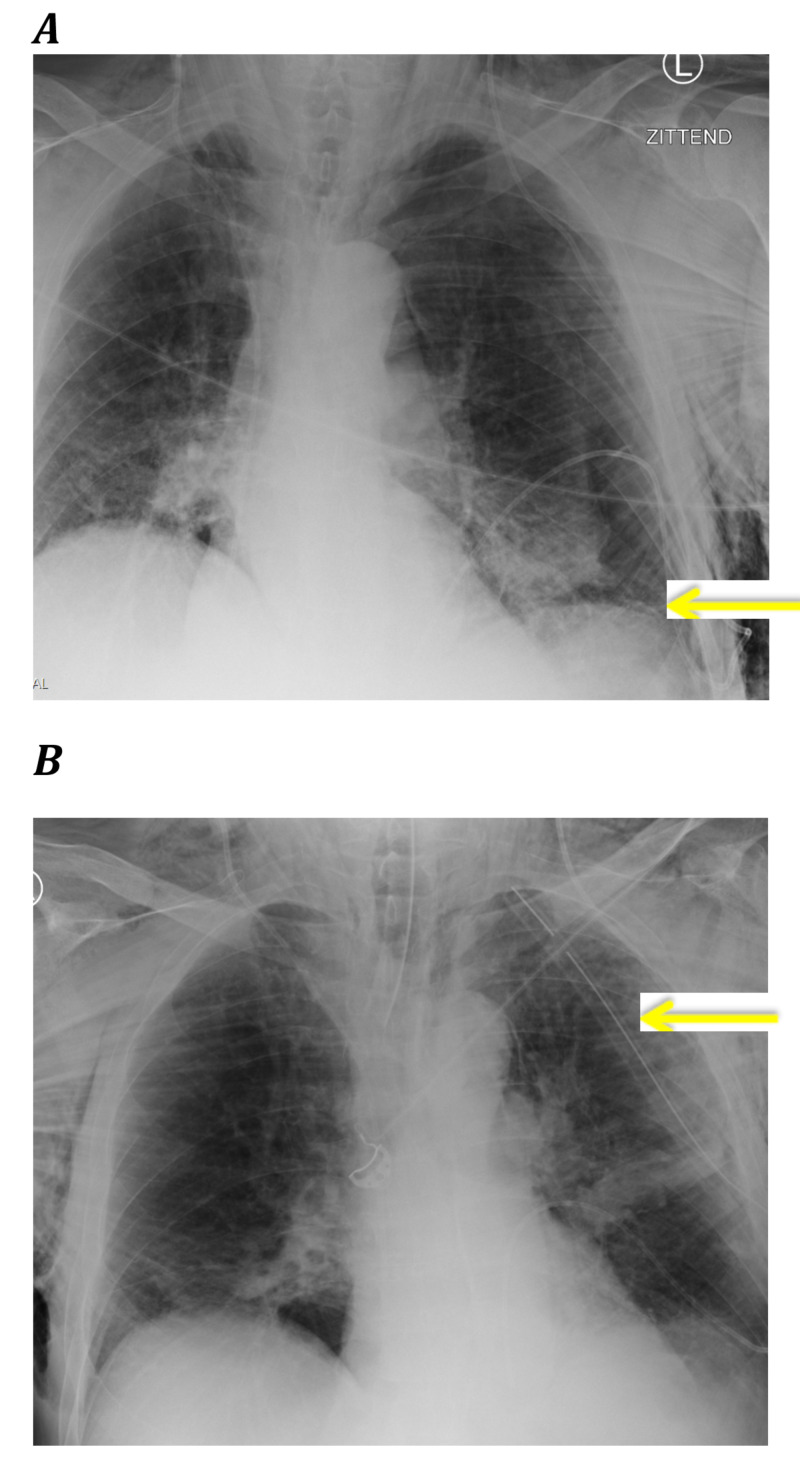
Chest X-rays directly after the surgery for the placement of the ventriculo-pleural drain, showing a pneumothorax of left lung. A: before thorax drain placement (the yellow arrow shows the pneumothorax) B: after thorax drain placement (the yellow arrow shows good placement of the thorax drain)

Exactly two weeks later, the neurosurgeons decided to place a ventriculo-pleural drain. The surgery itself was uneventful and the patient was extubated. Directly after the patient developed progressive dyspnea with an arterial oxygen saturation of 80%, he developed subcutaneous emphysema over his thorax. A chest X-ray was performed and showed a pneumothorax for which a chest tube was placed (Figure 1). After chest tube placement there was no respiratory improvement, so it was decided to re-intubate and to transfer the patient from the post-anesthesia care unit (PACU) to the ICU.

At the ICU the sedation was stopped and eventually the patient was extubated again. However, his pulmonary condition was not very sufficient. There was a significant amount of air leakage in the chest tube system and there was an increase in subcutaneous emphysema.

**Figure 2:**
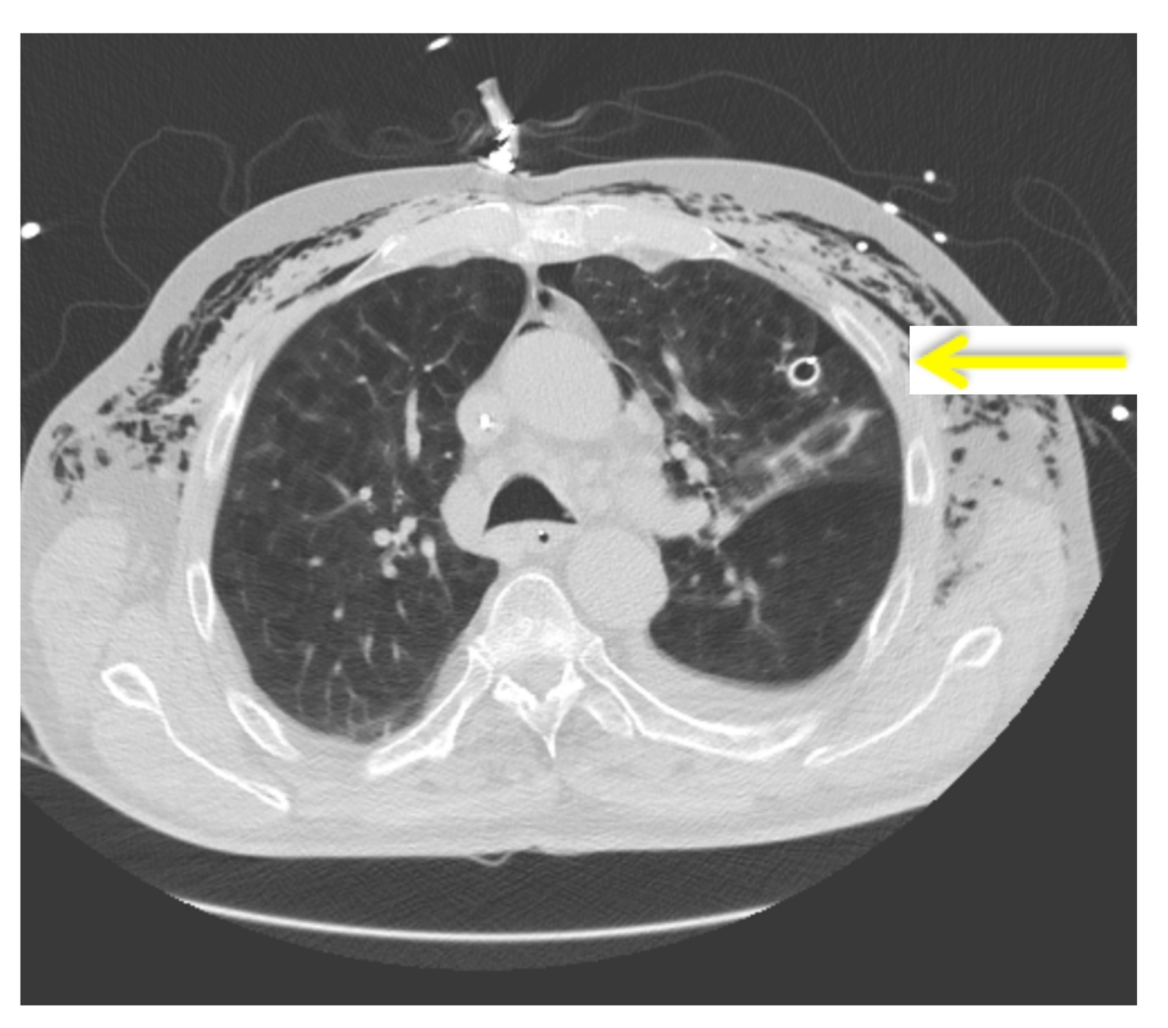
CT scan of the thorax showing the intrapleural location of the thorax drain. The yellow arrow indicated the intrapleural location of the thorax drain

**Figure 3:**
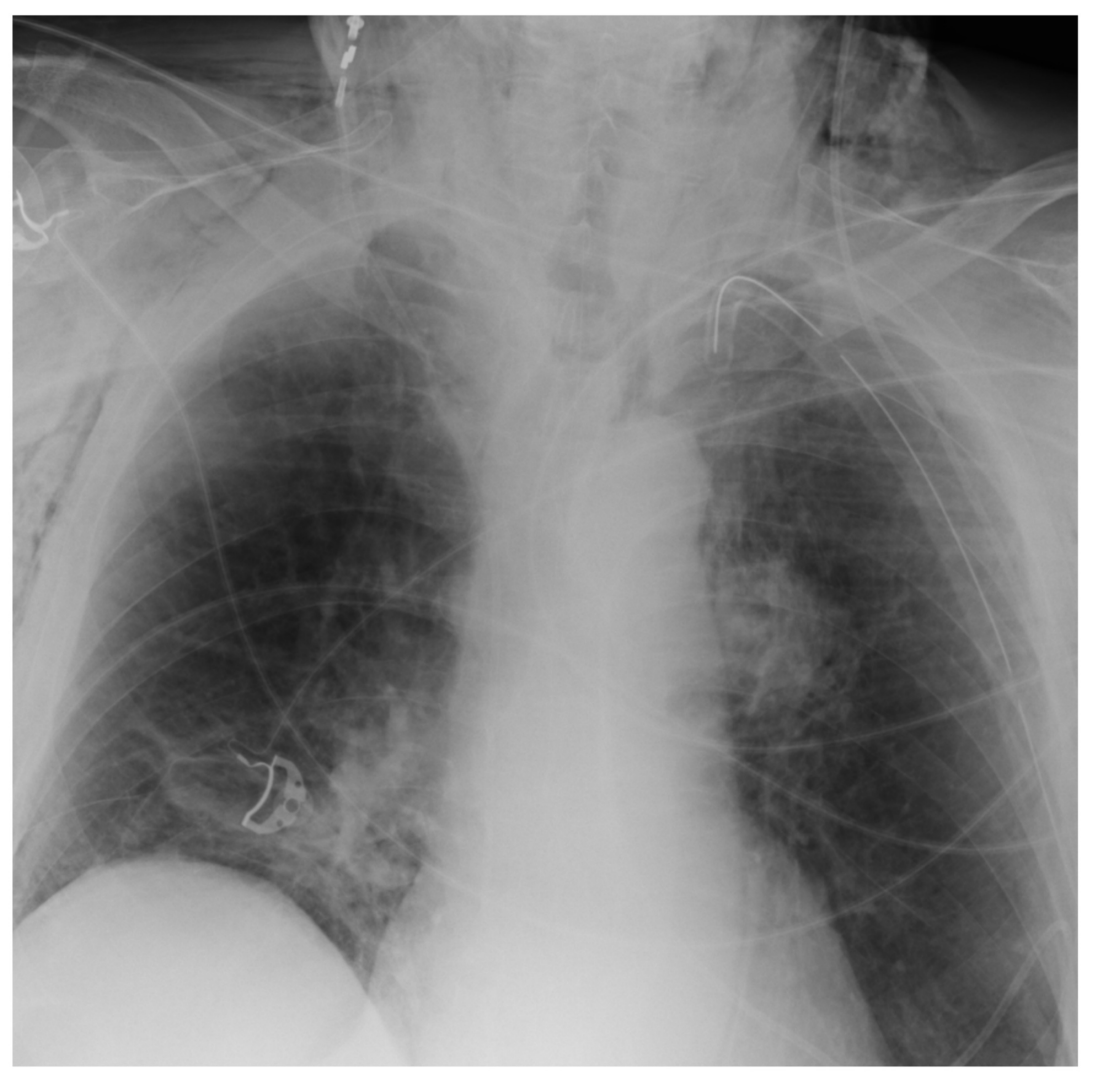
Postoperative chest X-ray after VATS wedge resection of the cranial part of the left upper lobe. VATS, video assisted thoracoscopic surgery

Therefore, a CT scan of the thorax was conducted and showed that the chest tube was placed in the lung parenchyma (Figure 2). Approximately one day later the patient underwent a video assisted thoracoscopic surgery (VATS). The leakage was found in the cranial tip of the left lung; however, initial attempts to staple it off were unsuccessful, so a wedge resection of the cranial part of the left upper lobe was done and a chest tube was placed. Figure 3 shows the postoperative chest X-ray. During the VATS, the neurosurgeon placed a Codman Hakim 30 mm-programmable valve in the ventriculo-pleural drain preventing backward flow of air.

**Figure 4:**
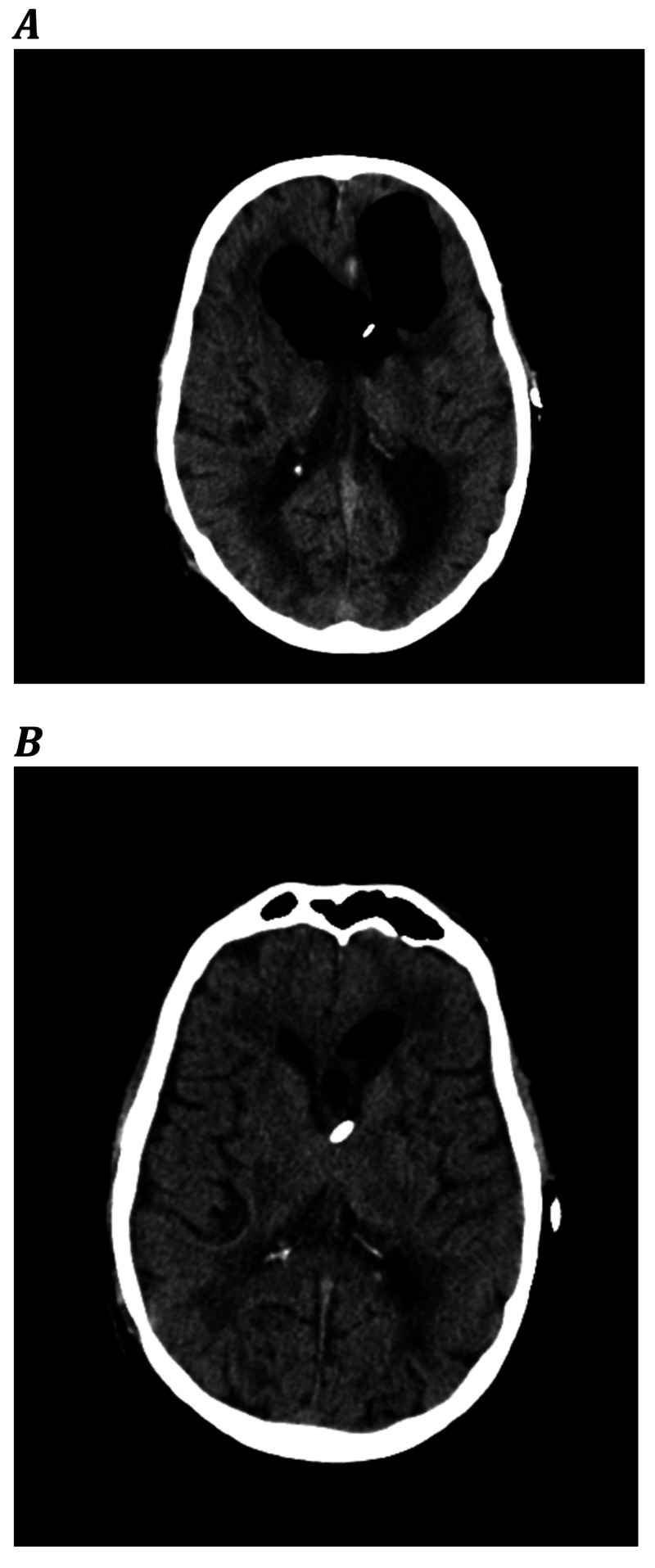
CT scans of the brain showing a pneumocephalus. A: before 100% oxygen treatment B: after 100% oxygen treatment

Shortly thereafter the patient developed very rigid extremities, a right-sided hemiparesis, anisocoric pupils, and a somnolent state. A CT scan of the brain was made showing a pneumocephalus. Other causes were ruled out; there were no signs of substance abuse withdrawal, no medication side effects (patient was not on haloperidol), and temperature was normal (37.4 degrees Celsius). We considered pneumocephalus-related catatonia. The BFCRS was used to assess the symptoms and severity of the catatonia (Table 1, T1). Because of the pneumocephalus treatment with 100% oxygen was started. Two days later he showed significant neurological improvement with a Glasgow Coma Scale of 13 (E4M6V3). Right after this improvement the BFCRS was used to reassess the situation and showed a significant clinical improvement (Table 1, T2). Figure 4 shows the brain CT scans before and after treatment.

**Table 1:** Assessment of catatonia using the BFCRS before (T1) and after treatment with 100% oxygen for pneumocephalus (T2). The method described here is used to complete the 23-item BFCRS and the 14-item CSI (the first 14 items of this table). Item definitions on the two scales are the same. The BFCRS measures the severity of 23 signs on a 0-3 scale, while the CSI measures only the presence or absence of the first 14 signs [10-11]. BFCRS, Bush-Francis Catatonia Rating Scale; CSI, Catatonia Screening Instrument

Items BFRCS	T_1_	T_4_
1. Immobility/stupor	2	0
2. Mutism	3	1
3. Staring	3	0
4. Posturing/catalepsy	1	0
5. Grimacing	3	1
6. Echopraxia/echolalia	0	0
7. Stereotypy	1	0
8. Mannerisms	0	0
9. Verbigeration	0	0
10. Rigidity	3	1
11. Negativism	0	0
12. Waxy flexibility	3	1
13. Withdrawal	0	0
14. Excitement	0	0
15. Impulsivity	0	0
16. Automatic obedience	0	0
17. Passive obedience	0	0
18. Muscle resistance	3	0
19. Motorically stuck	3	0
20. Grasp reflex	0	0
21. Perseveration	0	0
22. Combativeness	0	0
23. Automatic abnormality	2	0
Screening score	19	4
Severity score	27	4

Finally the patient developed an atrial flutter, for which he already had beta-blockers. The dosage was increased and the atrial flutter converted back to a sinus rhythm. Also a transthoracic echocardiogram (TTE) was performed that showed a good left and right ventricular function with a dilated right atrium (due to the earlier described lung emboli). A few days after discharge from the ICU, the patient went home without complications.

## Discussion

As described earlier in the literature and in our present case report, catatonia can be considered a very complex and dynamic clinical entity that is yet not fully understood [6]. As the original description and classification done by Kahlbaum in 1870s, there have been numerous efforts done by scientists and clinicians to upgrade and improve the definitions, classification and diagnosing tools, which eventually caused more doubt and inconsistencies [6-8]. However if the catatonia is diagnosed correctly, it is a treatable condition in most patients [7-8]. Our patient had catatonia due to reversal airflow to ventriculo-pleural drain. We also have to take into account that catatonia can be induced by solely psychiatric disorders or neurological disorders or a combination of both [6-8]. As our patient did not have a psychiatric medical history, but did have a 'damaged' brain due to previous TIAs and bleeding in the basal ganglia, we think that this form of catatonia has a merely neurological basis. He had also muscle spasm and atrial flutter, which could be part of this syndrome. His condition was treated in the ICU with good results.

Interestingly the most scientific articles on catatonia are case reports or case series, which makes consensus in treatment even more difficult [8-12]. In the recent literature it has been shown that catatonia is still highly prevalent and not restricted to schizophrenia spectrum disorders [8, 10, 12]. In our opinion, future research should focus on improving and forming consensus-based treatment protocols for patients with catatonia.

The definitions of catatonia are another side of the problem with a concurring 40 different signs and symptoms [8, 10, 12]. Of these symptoms most of them have a significant association with catatonia [7]. The original classification done by Kahlbaum had four groups of symptoms ranging from pure motor signs, disturbances of volition, inability to suppress complex motor activities, and autonomic instability [6-8]. Our patients had an atrial flutter which was treated successfully with beta-blockers and with no significant cardiologic pathology seen on the TTE. This atrial flutter can be considered part of symptom group ‘autonomic instability.’

Most of the times, symptoms associated with catatonia usually wax and wane sometimes within one hour [7-8]. Even though some are more prevalent than others, there is no single specific symptom to identify catatonia. Finally, the catatonic syndrome may become malignant with increased mortality particularly when autonomic instability is included [7-8]. The catatonia syndrome frequently occurs in schizophrenia spectrum disorders and affective disorders, but also in autism, dementia, intoxications, and in general medical conditions [8, 12-14]. The onset and duration of symptoms vary considerably.

The combination of pneumocephalus and (some sort of) catatonic behavior is very rare. To our knowledge this is the third case report that reports this rare combination. Kwon et al. reports a 57-year-old male who fell down from 3 m height, complicated by a tension pneumocephalus five months after trauma, with stupor and cognitive problems as initial symptoms [14]. Lutjens et al. reported a 56-year-old man with left hemianopsia who underwent surgical removal of a right parieto-occipital glioblastoma [13]. Six months after the initial surgery he presented with headache and a hygroma over the right hemisphere, for which a burr hole was made posterior to the right coronal suture. This was adequate to drain the subdural hygroma. Four hours after surgery he developed severe Parkinsonism and akinetic mutism due to a subdural and intraventricular tension pneumocephalus [13]. He was drained and got a subduro-peritoneal shunt. Afterwards his clinical symptoms improved. Akinetic mutism, catatonia, and Parkinsonism have many similarities and due to clinical heterogeneity they are difficult to set apart from each other and to adequately diagnose. A recent review done by Arnts and colleagues tried to summarize the neuropathophysiological and neuroanatomical localization for akinetic mutism and possible treatment strategies [15]. Despite advances made in recent years in understanding of the neuroanatomy, neuro-physiology, and chemoarchitecture of neurocircuits that are involved in the initiation and motivation of behavior, it is still not fully understood [15].

## Conclusions

Catatonia and pneumocephalus can be a challenging combination of diagnoses that might need multidisciplinary treatment. The combination of a catatonic syndrome and pneumocephalus in neurosurgical and intensive care practice is a rare phenomenon. One should consider it in patients after complicated neurosurgery with pneumocephalus.
